# Impact of influenza PA-X on host response

**DOI:** 10.18632/oncotarget.5127

**Published:** 2015-08-10

**Authors:** Tsuyoshi Hayashi, Chutikarn Chaimayo, Toru Takimoto

**Affiliations:** Department of Microbiology and Immunology, University of Rochester Medical Center, Rochester, New York, USA

**Keywords:** Immunology and Microbiology Section, Immune response, Immunity, influenza, shutoff, virus

Influenza virus infection causes global shutdown of host protein synthesis in the infected cells, while viral proteins are efficiently produced. This host shutoff activity is considered to allow the virus to escape host innate and acquired immune recognition, which otherwise restricts viral replication and spread. It had been thought that cap snatching activity and degradation of host RNA polymerase II induced by viral RNA polymerase cause host shutoff [1,2]. However, a recent study revealed that influenza A virus expresses an additional viral protein, PA-X that plays a major role in suppression of host protein synthesis in infected cells [3].

PA-X encodes N-terminal 191 amino acids of PA protein and unique C-terminal sequences (41 or 61 amino acid residues) produced by +1 reading frame of PA mRNA via ribosomal frameshifting [3]. PA-X contains endonuclease active domain in its N-terminal region that is common to the PA protein. We and Jagger et al., demonstrated that transfection of a PA-X variant containing a single mutation at the endonuclease active site failed to suppress production of co-expressed reporter proteins, indicating that PA-X suppresses host protein synthesis via mRNA decay [3,4]. Although both PA and PA-X have identical endonuclease active site in their N-terminal domain, PA-X induced much stronger host shutoff activity than PA, suggesting an important role of unique C-terminal region of PA-X in host shutoff [3,4]. A recent report indicates that six basic amino acid residues within the N-terminal 15 residues of PA-X-unique region played a role in the optimal shutoff activity [5]. These basic residues are highly conserved among influenza A viruses, suggesting their essential role in effective shutoff activity [5]. PA-X activities in host shutoff vary among strains. Residues within the common N-terminal domain reflected the difference in PA-X activity [4]. Interestingly, PA-X from avian viruses was more active than that of human viruses, which might suggest a role of PA-X in optimal growth in their specific hosts [4].

To analyze the effect of PA-X of currently circulating viruses on virus growth and pathogenicity, we recently rescued a mutant 2009 pandemic H1N1 virus (pH1N1) expressing reduced amount of PA-X through mutations at the frameshift motif (PA-XFS) [6]. In agreement with the results using 1918 highly pathogenic influenza A virus [3], pH1N1 PA-X degrades host mRNA and suppresses host protein synthesis in infected cells [6]. Unlike 1918 PA-XFS, however, pH1N1 PA-XFS was attenuated both in cultured human cells and the infected mouse lungs [6]. Attenuated virus growth was accompanied with stronger IFN-β response *in vitro* and *in vivo*. The pH1N1 PA-XFS-infected mice cleared the virus and recovered faster than wild type-infected mice [6]. Given the fact that PA-X suppresses expressions of MHC class I-associated genes in infected mouse lungs [3], it is possible that PA-X inhibits proper antigen presentation that allows efficient viral clearance. Influenza A viruses express NS1 protein, which specifically targets and blocks host innate immunity. Although we detected attenuated phenotype of pH1N1 PA-XFS, the impact of PA-X on virus growth may vary between the virus strains depending on the specificity and activity of NS1 protein of each strain.

Highly pathogenic influenza viruses, such as 1918 virus or avian H5N1 viruses induce an extensive host cytokine response, known as cytokine storm, causing tissue injury and virus pathogenicity. Reduced PA-X expression of 1918 virus enhanced virus pathogenicity due to the accelerated cytokine responses and severe tissue injury [3]. Avian H5N1 PA-XFS also showed increased pathogenicity in avian species through the induction of excessive host inflammatory gene expressions [7]. In the case of pH1N1 virus, impact of PA-X in viral pathogenicity was less evident than that of highly pathogenic viruses as measured by lethal dose, although greater degrees of inflammatory cell infiltration were observed in the infected mouse lungs [6]. These data highlight the strong impact of PA-X in viral pathogenicity, especially for highly pathogenic viruses that induce excessive cytokine response upon infection.

Importantly, pH1N1 PA-XFS induced a greater humoral response than wild type virus in mice, even though virus replication was attenuated in their lungs [6]. This result indicates that PA-X also affects the acquired immune response and antibody production. Consistent with our data, global transcriptional profiling of the infected mouse lungs showed difference in expression of immune regulatory genes between 1918 wild type and PAXFS [3]. Seasonal influenza A viruses induce relatively weak immune response, causing recurrent infections. General shutoff of host gene expression by PA-X likely contributes to virus escape from immune recognition and appropriate antigen processing to induce acquired immunity. Our data suggest that the efficacy of live attenuated vaccines could be improved by suppressing PA-X expression. Considering the strong impact of PA-X in immune response, further analysis on the role of PA-X in immune modulation is required to unveil the mechanism of viral immune evasion, which will lead to the development of effective vaccines against influenza infection.
